# Rituximab in PR3-ANCA positive patients with moderately to severely active ulcerative colitis: a multicenter real-world pilot study

**DOI:** 10.3389/fphar.2025.1621795

**Published:** 2025-10-24

**Authors:** Qi Yu, Yiyu Cheng, Huan Wang, Yidong Chen, Fang Liu, Xiaopeng Zhang, Xiaoyu Fu, Jiamin Li, Junrong Li, Ying Li, Liangru Zhu

**Affiliations:** ^1^ Department of Gastroenterology, Union Hospital, Tongji Medical College, Huazhong University of Science and Technology, Wuhan, China; ^2^ Department of Gastroenterology, The First Affiiated Hospital of Shihezi University, Shihezi, China

**Keywords:** PR3-ANCA, UC, rituximab, effectiveness, real-world study

## Abstract

**Background and aims:**

Patients with ulcerative colitis (UC) and PR3-ANCA positivity often show a poor response to infliximab (IFX). Our objective was to compare the effectiveness and safety of rituximab (RTX) and IFX in moderately-to-severely active PR3-ANCA positive (PR3-ANCA+) UC patients. This study represents the first exploration of biomarker-guided therapy (PR3-ANCA+) in moderately-severely active UC, aiming to generate hypothesis-driven evidence for future randomized trials.

**Methods:**

This retrospective, multicenter, real-world study focused on a rare biomarker-defined subgroup (PR3-ANCA + UC) and was conducted across three medical centers in Hubei, China. Moderately to severely active UC patients with PR3-ANCA+ were assigned to the RTX group (n = 12) and the IFX group (n = 26) based on biological therapy received. Endpoints at week 22 included clinical remission (primary), clinical response, endoscopic response, improvement, and remission. Safety endpoints centered on opportunistic infections and drug-related adverse events. Inverse probability of treatment weighting (IPTW) and multifactorial logistic regression analysis (MLRS) were used to reduce confounding effects.

**Results:**

Pre-IPTW, compared with IFX, RTX significantly increased the rates of clinical remission, clinical response, endoscopic response, endoscopic improvement, and endoscopic remission by 60.25% (95%CI: 33.68%–86.82%, *P* < 0.001), 34.62% (95%CI: 16.33%–52.91%, *P* = 0.020), 65.38% (95%CI: 45.15%–85.61%, *P* < 0.001), 38.46% (95%CI: 10.65%–66.27%, *P* = 0.010), and 65.38% (95%CI: 45.15%–85.61%, *P* < 0.001), respectively. After IPTW, RTX remained associated with significantly higher rates of the above outcomes versus IFX, with increases of 53.68% (95%CI: 35.26%–72.10%, P = 0.012), 33.90% (95%CI: 16.52%–51.28%, *P* = 0.002), 62.71% (95%CI: 46.35%–79.07%, *P* < 0.001), 46.61% (95%CI: 26.83%–66.39%, *P* = 0.024), and 62.71% (95%CI: 46.35%–79.07%, *P* < 0.001), respectively. In MLRS, RTX was associated with higher odds of week-22 clinical remission compared with IFX, with consistent results before and after IPTW [pre-IPTW: OR = 31.022 (2.911–970.983), *P* = 0.010; post-IPTW: OR = 47.692 (4.077–1,418.298), *P* = 0.007]. Additionally, for every 50% reduction in PR3-ANCA levels, the odds ratio (OR) for clinical remission was 7.583 (95%CI: 1.648–34.903). Furthermore, this conclusion remained robust after adjusting for confounding factors. For safety endpoints, no RTX patients had elevated tuberculosis interferon tests, while 2 IFX patients (7.69%) did. In addition, no HBV reactivation or infection occurred in either group. Mean IgG levels remained stable in RTX-treated patients (11.50 ± 3.59 vs. 10.84 ± 1.48, *P* = 0.569).

**Conclusion:**

In moderately to severely active UC patients with PR3-ANCA+, RTX showed better effectiveness than infliximab (IFX), with a similar safety profile.

## 1 Introduction

Ulcerative colitis (UC), an inflammatory bowel disease, exerts a substantial negative influence on the quality of life of patients, often leading to long-term burdensome complications (Le Berre et al., 2023). Epidemiological data reveal a sharp increase in incidence rates in both low/middle-income countries (Kaplan, 2015; Ng et al., 2017) and several high-income nations, such as Finland (Kontola et al., 2023), Denmark (Lophaven et al., 2017), and the United States (Keyashian et al., 2019), highlighting UC as a global health challenge. According to the ECCO guidelines, infliximab (IFX) is recommended for inducing remission in moderate-to-severe UC patients who show an inadequate response or have an intolerance to conventional therapies (Raine et al., 2022). However, some patients with anti-neutrophil cytoplasmic antibody positivity (ANCA+) show poor responsiveness to IFX (Ferrante et al., 2007; Jürgens et al., 2010; Yoshida et al., 2021). Hence, developing a new advanced therapy that does not compromise safety is an urgent need to improve the treatment status of moderate-to-severe UC patients with ANCA+.

The growing comprehension of the functions played by B cell response (Uzzan et al., 2022) and ANCA (Calderón-Gómez and Panés, 2012) indicates that strategies aimed at eliminating B-cell might serve as a viable therapeutic approach for UC, especially for those patients with ANCA+. Prior clinical evidence has demonstrated an association between ANCA+ and more severe manifestations of UC (Mahler et al., 2013; Takedatsu et al., 2018; Aoyama et al., 2021; Imakiire et al., 2022; Wen et al., 2022; Zeng et al., 2023), yet the precise mechanisms remain elusive. Speeding up the elimination of serum ANCA so as to decrease the generation of colonic Neutrophil Extracellular Traps (NETs), a potential factor in sustaining mucosal inflammation in UC, might be beneficial for treatment strategies (Wen et al., 2022). These findings suggest that ANCA may exacerbate the mucosal inflammation caused by UC (Aoyama et al., 2021). Consequently, strategies for B-cell deletion are expected to emerge as a successful therapeutic alternative for UC patients.

Rituximab (RTX), a humanized anti-CD20 chimeric monoclonal antibody, accurately targets the CD20 antigen located on the surface of B lymphocytes and attaches to it, ultimately causing a reduction in the number of CD20-positive B cells (Tedder and Engel, 1994; Riley and Sliwkowski, 2000). Nevertheless, a research has shown that in patients with moderately active UC who have an insufficient response to oral steroids, RTX fails to induce remission (Leiper et al., 2011). It’s important to note, though, that this study has several limitations: 1) The ANCA status of patients is yet to be determined, and this might potentially cause an underestimation of the effectiveness of RTX; 2) The administered dose of RTX might have been excessive—a single dose of 1,000 mg could have contributed to disease exacerbation; 3) There’s uncertainty regarding the state of B cell depletion due to a lack of monitoring; 4) The analysis relied on limited sample size, and this circumstance has the potential to undermine the reliability of the outcomes.

This research is targeted at contrasting the effectiveness and safety of RTX and IFX among ANCA + patients with moderately to severely active UC. Considering that cases of proteinase 3 -ANCA+ (PR3-ANCA+) UC are widespread in Asian regions (Xu et al., 2008; Takedatsu et al., 2018), this study focuses on patients with PR3-ANCA + UC as the target population.

## 2 Materials and methods

### 2.1 Study design

This retrospective, multicenter, real-world study was conducted between May 2020 and September 2023 across three medical centers in Hubei, China: Huazhong University of Science and Technology Tongji Medical College-affiliated Union Hospital, Shiyan Taihe Hospital, and Jingzhou First People’s Hospital. Moderately to severely active UC patients with PR3-ANCA+ were assigned to either the RTX or IFX group based on their biological therapy. Data extraction was completed in June 2024. Eligibility criteria for patients included: (1) Age ≥18 years; (2) A definitive diagnosis of UC; (3) Mayo Clinic Score (MCS) of 6–12; (4) PR3-ANCA+ with levels exceeding 20 CU as confirmed by both indirect immunofluorescence and enzyme-linked immunosorbent assay; (5) Complete baseline clinical data and following outcomes; (6) Treatment with RTX or IFX to induce remission due to an inadequate response, loss of response, or intolerance to previous treatment such as immunosuppressant, biological drugs, or corticosteroid treatment. Patients were excluded if they: (1) were undergoing treatment with other biologics or small molecule drugs; (2) had previous intestinal surgery, such as colectomy, ileostomy, or colostomy; (3) Had infections such as HIV, hepatitis B/C virus, cytomegalovirus, tuberculosis, or others; (4) Did not have endoscopic examination results at week 22. Consequently, 38 patients were enrolled in the study.

### 2.2 PR3-ANCA and B Cell depletion evaluation

#### 2.2.1 Methodology for PR3-ANCA evaluation

As initially stated, PR3-ANCA positivity was defined as levels exceeding 20 CU (Clinical Units); the specific detection and confirmation procedures were as follows: (1) Indirect Immunofluorescence: Ethanol-fixed human neutrophils were used as the substrate. Serum samples were diluted at a 1:20 ratio and incubated with the substrate; after washing, a fluorescein isothiocyanate (FITC)-conjugated goat anti-human IgG secondary antibody was added for visualization. Positivity was determined by the presence of a cytoplasmic ANCA pattern (diffuse granular fluorescence throughout the neutrophil cytoplasm, with enhanced fluorescence in the nuclear lobes), which is characteristic of PR3-ANCA. (2) Enzyme-Linked Immunosorbent Assay: Recombinant human proteinase 3 (rPR3) was coated as the capture antigen. Serum samples were added to bind with rPR3, followed by incubation with a horseradish peroxidase (HRP)-conjugated anti-human IgG antibody and a chromogenic substrate (tetramethylbenzidine, TMB). The absorbance was measured at 450 nm, and PR3-ANCA levels (in CU) were calculated using a standard curve calibrated with international reference standards. A value >20 CU was confirmed as positive.

#### 2.2.2 Methodology for B Cell depletion evaluation

To evaluate B cell depletion (a key pharmacodynamic endpoint for rituximab treatment), B lymphocyte subsets were detected using multiparameter flow cytometry. Peripheral venous blood (3–5 mL) was collected from patients into EDTA-K2 anticoagulant tubes. Within 2 h of collection, samples were processed by lysing red blood cells with BD FACS Lysing Solution, centrifuging at 1,500 × g for 5 min, and resuspending the cell pellet in PBS containing 1% BSA to a concentration of 1 × 10^6^ cells/mL. The cells were then stained with fluorochrome-conjugated monoclonal antibodies (anti-CD45-FITC for leukocyte gating, anti-CD19-PE and anti-CD20-APC as B cell-specific markers) and analyzed on a BD FACSCanto II flow cytometer, with data processed using FlowJo 10.8.1 software.

### 2.3 Procedures

After completing a 7-day course of intravenous corticosteroids, the RTX group received either 100 mg or 200 mg of intravenous RTX with a 7-day interval between treatments. The total dose and dosing intervals of RTX are determined based on B lymphocyte levels and clinical symptoms. Achieving a complete depletion of the B lymphocyte subset is a crucial determinant in deciding whether to continue treatment with RTX. The general dosage range for RTX was 400 mg–900 mg. For the IFX group, a 5 mg/kg intravenous dose of IFX was injected at weeks 0, 2, and 6. After that, maintenance doses were administered every 8-week cycle Concomitant medications for all patients included 5-aminosalicylic acid (5-AA) and corticosteroids. Details are as follows: (1) 5-AA: oral, 2 g/day. (2) Corticosteroid: ① Intravenous corticosteroid until day 7; ② Oral prednisone was administered at a daily dose of 30 mg. During the first week, the amount was reduced by 10 mg, reaching 20 mg per day. After that, a weekly reduction of 5 mg was implemented until the treatment was discontinued.

### 2.4 Data sources and outcomes

All data, encompassing both baseline characteristics and follow-up outcomes, were extracted from medical records. The MCS and the Mayo Endoscopic Subscore (MES) were assessed by two senior physicians from the Department of Gastroenterology, based on endoscopic findings.

The main and secondary effectiveness endpoints measured at week 22 encompassed clinical remission (the primary endpoint), clinical response, endoscopic response, endoscopic improvement, and endoscopic remission. The definitions of these effectiveness endpoints are available in Supplementary Table S1.

Safety endpoints were concentrated on monitoring opportunistic infections, such as tuberculosis, cytomegalovirus (CMV), Epstein-Barr virus (EBV), *Clostridium difficile*, and bacterial infections, as well as adverse reactions related to the drugs.

### 2.5 Statistical analysis

Statistical analysis was conducted using SPSS 22.0 software (IBM Corporation, Armonk, NY, United States). Continuous data were reported as the mean ± standard deviation ( $\bar{x}$ ± SD) or interquartile range (IQR), while categorical data were presented as percentages (%). Group comparisons were made using the Wilcoxon test, Chi-square test, or Fisher’s exact test (when the minimum theoretical frequency was <5).

To account for potential confounding factors between the two treatment groups and ensure the reliability and rigor of the clinical outcomes, we utilized multifactorial logistic regression analysis and inverse probability of treatment weighting (IPTW). The IPTW was based on a multinomial propensity score (PS) model. Covariates of the PS model were identified *a priori*, based on current literature reviews and hypotheses about potential confoundings (Elhag et al., 2022; Dalal et al., 2024). The following preadmission confounders were included: age (>60 years), sex, BMI (<18.5 kg/m^2^), disease duration, disease extent (>proctitis), Mayo endoscopic subscore (MES) ≥2, PR3-ANCA levels, >1 advanced therapy class failure (biologic or small molecule), and concurrent oral prednisone. In addition, We consider Standardized Mean Differences (SMD) < 0.3 as acceptable for balance (Stuart et al., 2013; Song et al., 2021).

To investigate the relationship between B-cell depletion, PR3-ANCA levels, and clinical remission, we first calculated the B-cell reduction rate [formula: (baseline B lymphocyte level − B-cell level at week 22)/baseline B-cell level] and PR3-ANCA reduction rate [formula: (baseline PR3-ANCA level − PR3-ANCA level at week 22)/baseline PR3-ANCA level]. These reduction rates were then converted into categorical variables using 50% as the cutoff, and a binary Logistic regression model was applied to analyze their association with clinical remission.

## 3 Results

### 3.1 Baseline characteristics

**FIGURE 1:**
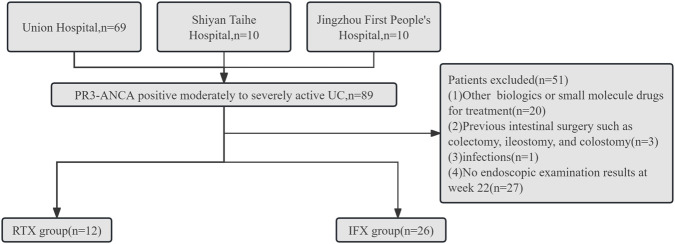
Workflow of the study.

Between May 2020 and September 2023, a total of 38 patients with moderately to severely active UC with PR3-ANCA+ were enrolled and assigned to the RTX group (n = 12) and the IFX group (n = 26). The workflow of the study is depicted in Figure 1.

In the original cohort, baseline characteristics were generally well-balanced between the RTX and IFX groups except for prior advanced therapies of Tofacitinib and IFX and >1 advanced therapy class failure (biologic or small molecule) (Table 1).

**TABLE 1:** Baseline characteristics between RTX and IFX groups.

Baseline characteristics	Original cohort				IPTW cohort^a^			
	RTX group (n = 12)	IFX group (n = 26)	P	SMD	RTX group (n = 35.4)	IFX group (n = 35.4)	P	SMD
Age (years)	37.17 ± 12.57	42.15 ± 15.01	-	-	-	-	-	
Age-binary variable			0.935	0.219			0.315	0.282
≥60 years	1 (8.33)	4 (15.39)			1.3 (3.67)	4.3 (12.15)		
<60 years	11 (100.00)	22 (84.61)			34.1 (96.33)	31.1 (87.85)		
Sex			1.000	0.077			0.950	0.026
Female	6 (50.00)	12 (46.15)			16.1 (45.48)	15.7 (44.35)		
Male	6 (50.00)	14 (53.85)			19.3 (54.52)	19.7 (55.65)		
BMI (Kg/m^2^)	19.28 (17.83, 20.17)	21.33 (18.65,23.84)	-	-	-	-	-	
BMI-binary variable			0.430	0.405			0.628	0.193
<18.5 Kg/m^2^	5 (41.67)	6 (23.08)			11.3 (31.92)	8.2 (23.16)		
≥18.5 Kg/m^2^	7 (58.33)	20 (76.92)			24.1 (68.08)	27.2 (76.84)		
Disease duration (Months)	14.50 (6.25, 126.00)	30.00 (9.00,72.00)	-	-	-	-	-	
Disease duration-binary variable			1.000	0.129			0.739	0.146
>5 years	3 (25.00)	8 (30.77)			8.4 (23.73)	10.8 (30.51)		
≤5 years	9 (75.00)	18 (69.23)			27 (76.27)	24.6 (69.49)		
Current cigarette smoker	0 (0.00)	1 (3.85)	1.000	0.283	0 (0.00)	1 (3.85)	0.332	0.243
Disease activity and severity								
MCS category			0.408	0.423			0.637	0.197
6∼10 (moderately)	4 (33.33)	14 (53.85)			16 (45.20)	19.4 (54.80)		
11∼12 (severely)	8 (66.67)	12 (46.15)			19.4 (54.80)	16 (45.20)		
Disease extent > proctitis	12 (100.00)	26 (100.00)	-	<0.001	35.4 (100.0)	35.4 (100.0)	-	<0.001
MCS	11.00 (9.75, 11.00)	11.00 (9.25, 11.00)	0.525	0.259	10.16 ± 1.11	9.84 ± 1.61	0.537	0.233
MES	3.00 (3.00, 3.00)	3.00 (2.50, 3.00)	0.555	0.213	2.89 ± 0.32	2.79 ± 0.42	0.467	0.285
MES-binary variable			-				-	<0.001
≥2	12 (100.00)	26 (100.00)		<0.001	35.4 (100.00)	35.4 (100.00)		
<2	0 (0.00)	0 (0.00)			0 (0.00)	0 (0.00)		
Biomarkers								
ALB			0.982	0.161			0.815	0.095
≤40 mg/mL	2 (16.67)	6 (23.08)			6.6 (18.64)	8 (22.60)		
>40 mg/mL	10 (83.33)	20 (76.92)			28.8 (81.36)	27.4 (77.40)		
FC			1.000	0.045			0.810	0.103
≤100 µg/g	3 (25.00)	7 (26.92)			11.9 (33.62)	10.2 (28.81)		
>100 µg/g	9 (75.00)	19 (73.08)			23.5 (66.38)	25.2 (71.19)		
CRP			1.000	0.044			0.673	0.179
≤5 mg/L	3 (25.00)	7 (26.92)			7.9 (22.32)	10.6 (29.94)		
>5 mg/L	9 (75.00)	19 (73.08)			27.5 (77.68)	24.8 (70.06)		
PR3-ANCA(CU)	143.94 ± 118.87	108.41 ± 130.24	0.643	0.166	148.44 ± 117.45	128.56 ± 131.77	0.688	0.159
B lymphocytes (%)	12.45 ± 8.07	-	-		-	-	-	
Prior advanced therapies								
Tofacitinib	3 (25.00)	0 (0.000)	0.008	-	-	-	-	-
IFX	6 (50.00)	2 (7.69)	0.007	-	-	-	-	-
VDZ	5 (41.67)	6 (23.08)	0.272	-	-	-	-	-
Prior advanced therapies-binary variable: >1 advanced therapy class failure (biologic or small molecule)	5 (41.67)	1 (3.85)	0.013	1.011	29.5 (83.33)	32.3 (91.24)	0.552	0.234
Concurrent medications								
Oral prednisone	12 (100.00)	26 (100.00)	-	<0.001	-	-	-	<0.001
5-Aminosalicylic Acid	12 (100.00)	26 (100.00)	-	<0.001	-	-	-	<0.001

^a^ Adjusted covariates in IPTW cohort: age, sex, BMI, disease duration, disease extent (>proctitis), MES≥2, PR3-ANCA, levels, >1 advanced therapy class failure (biologic or small molecule), and concurrent oral prednisone. All included covariates are binary variables, except PR3-ANCA levels.

Abbreviations: RTX, rituximab; IFX, infliximab; BMI, body mass index; MCS, mayo clinic score; MES, mayo endoscopic subscore; FC, fecal calprotectin; CRP, C-reactive protein; ALB, albumin.

Since disease extent > proctitis and MES ≥2 are present in 100% of both groups, the PS model consisted of Age, Sex, BMI, Disease duration, >1 advanced therapy class failure. All included covariates are binary variables, except PR3-ANCA levels. The inverse probability of the IPTW cohort demonstrated that between-group imbalances were eliminated, as shown in Table 1.

### 3.2 Details of B lymphocytes and PR3-ANCA levels in patients treated with RTX

**FIGURE 2:**
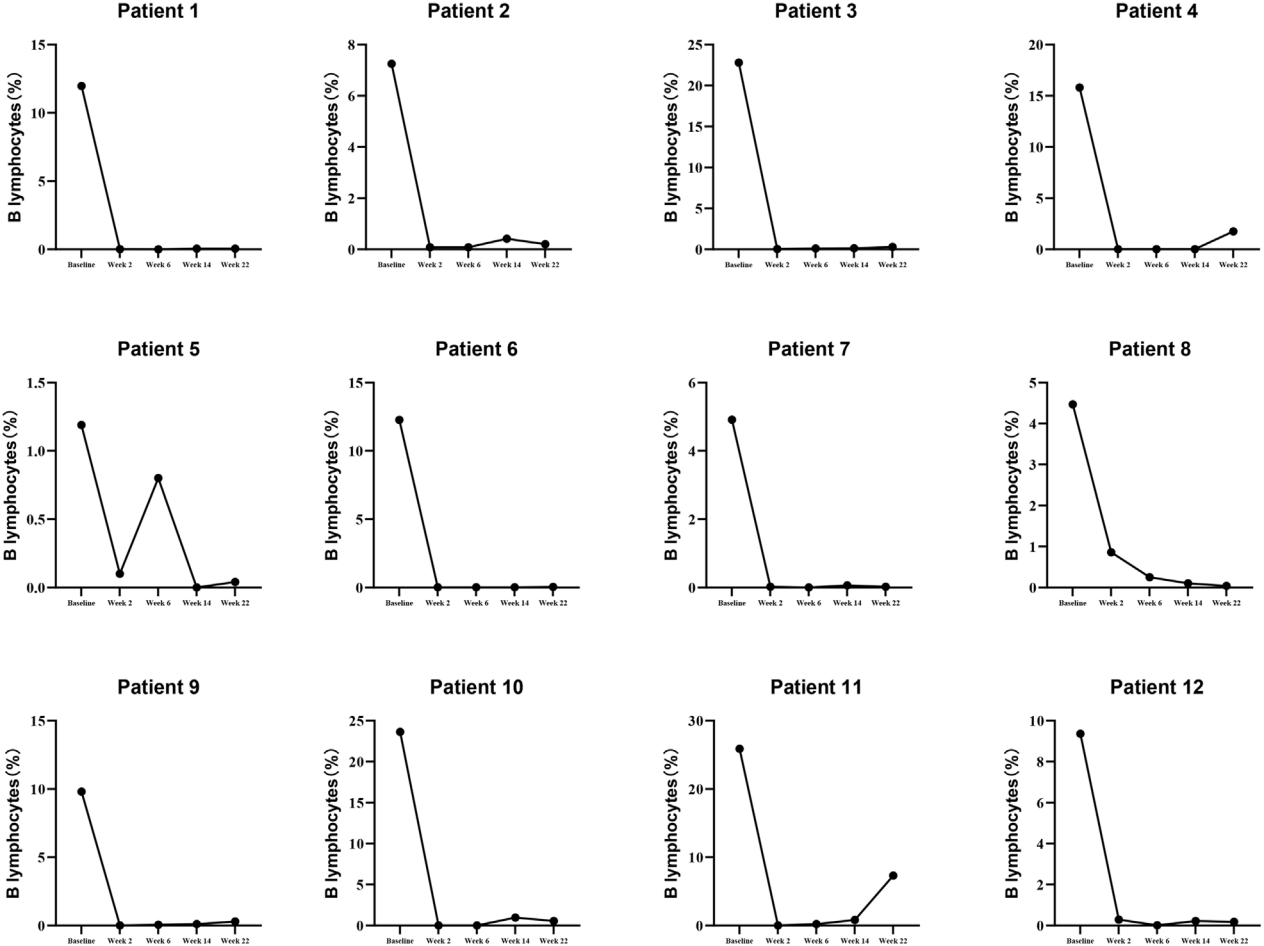
The details of B lymphocytes in patients receiving RTX Therapy.

**FIGURE 3:**
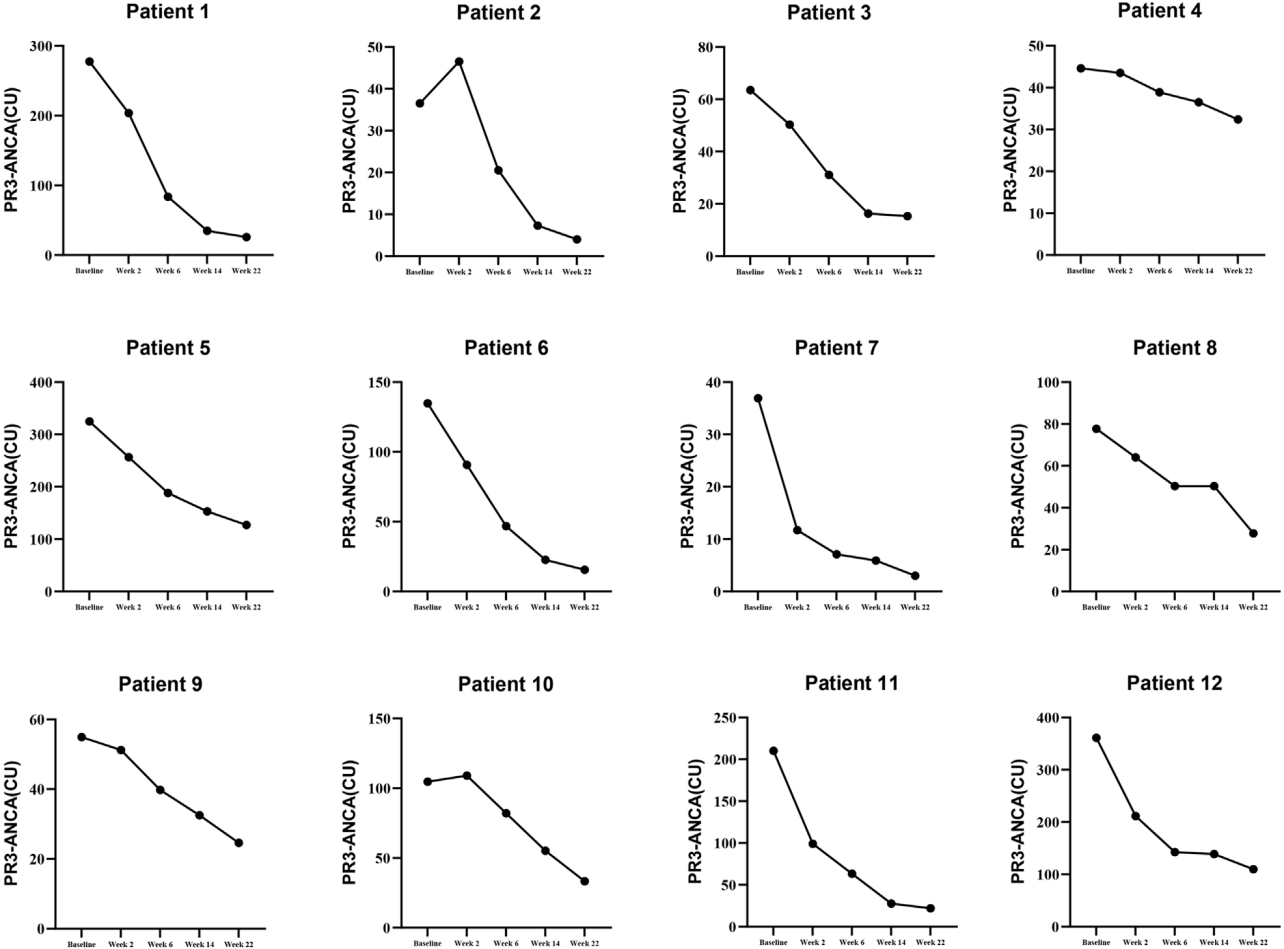
The details of PR3-ANCA in patients receiving RTX T.herapy.

To illustrate the details of RTX (Rituximab) treatment, this study presents the levels of B lymphocytes and PR3-ANCA at baseline, week 2, week 6, week 10, week 14, and week 22, as detailed in Supplementary Table S2 and depicted in Figures 2, 3. Furthermore, the effectiveness endpoints for patients treated with RTX at week 22 are outlined in Supplementary Table S3.

### 3.3 Effectiveness endpoints analyses

In the original cohort, higher proportions of patients treated with RTX achieved the following outcomes versus IFX-treated patients: clinical remission [10 (83.33%) vs. 6 (23.08%), *P* < 0.001], clinical response [12 (100.00%) vs. 17 (65.38%), *P* = 0.020], endoscopic response [12 (100.00%) vs. 9 (34.62%), *P* < 0.001], endoscopic remission [6 (50.00%) vs. 3 (11.54%), *P* = 0.010], and endoscopic improvement [12 (100.00%) vs. 9 (34.62%), *P* < 0.001] Specifically, pre-IPTW, compared with IFX, RTX significantly increased the rates of clinical remission, clinical response, endoscopic response, endoscopic improvement, and endoscopic remission by 60.25% (95%CI: 33.68%–86.82%), 34.62% (95%CI: 16.33%–52.91%), 65.38% (95%CI: 45.15%–85.61%), 38.46% (95%CI: 10.65%–66.27%), and 65.38% (95%CI: 45.15%–85.61%), respectively. After IPTW, RTX remained associated with significantly higher rates of the above outcomes versus IFX, with increases of 53.68% (95%CI: 35.26%–72.10%), 33.90% (95%CI: 16.52%–51.28%), 62.71% (95%CI: 46.35%–79.07%), 46.61% (95%CI: 26.83%–66.39%), and 62.71% (95%CI: 46.35%–79.07%), respectively. Details are shown in Table 2.

**TABLE 2:** Effectiveness endpoints at week 22 between RTX and IFX groups.

Effectiveness endpoints at week 22	Original cohort				IPTW cohort^a^			
	RTX group (n = 12)	IFX group (n = 26)	Risk differences (95%CI)	*P*	RTX group (n = 35.4)	IFX group (n = 35.4)	Risk differences (95%CI)	*P*
Clinical remission	10 (83.33)	6 (23.08)	60.25% (33.68%–86.82%)	<0.001	28.8 (81.36)	9.8 (27.68)	53.68% (35.26%–72.10%)	0.012
Clinical response	12 (100.00)	17 (65.38)	34.62% (16.33%–52.91%)	0.02	35.4 (100.00)	23.4 (66.10)	33.90% (16.52%–51.28%)	0.002
Endoscopic response	12 (100.00)	9 (34.62)	65.38% (45.15%–85.61%)	<0.001	35.4 (100.00)	13.2 (37.29)	62.71% (46.35%–79.07%)	<0.001
Endoscopic remission	6 (50.00)	3 (11.54)	38.46% (10.65%–66.27%)	0.01	22.6 (63.84)	6.1 (17.23)	46.61% (26.83%–66.39%)	0.024
Endoscopic improvement	12 (100.00)	9 (34.62)	65.38% (45.15%–85.61%)	<0.001	35.4 (100.00)	13.2 (37.29)	62.71% (46.35%–79.07%)	<0.001

^a^ Adjusted covariates in IPTW cohort: age, sex, BMI, disease duration, disease extent (>proctitis), MES≥2, PR3-ANCA levels, >1 advanced therapy class failure (biologic or small molecule), and concurrent oral prednisone. All included covariates are binary variables, except PR3-ANCA levels.

MLRS were adjusted for covariates including age-binary variable, sex, BMI-binary variable, disease duration-binary variable, current cigarette smoker, MCS category, Disease extent > proctitis, MCS, MES-binary variable, ALB, FC, CRP, and >1 advanced therapy class failure (biologic or small molecule). Compared to IFX, RTX was linked to substantially greater odds of achieving clinical remission within the original cohort [143.854 (1.060–19521.653), *P* = 0.047] and IPTW cohort [190.499 (4.147–1.000*10^5^), *P* = 0.032]. Additionally, RTX showed non-significantly different odds for clinical response, endoscopic response, endoscopic remission, and endoscopic improvement compared to IFX (all *P* > 0.05), as shown in Table 3.

**TABLE 3:** Multivariate analyses of effectiveness endpoints before and after IPTW.

Multivariate analyses^a^	Original cohort		IPTW cohort^b^	
	OR (95%CI)	P	OR (95%CI)	P
Clinical remission				
IFX	-	Ref.	-	Ref.
RTX	143.854 (1.060–19521.653)	0.047	190.499 (4.147–1.000*10^5^)	0.032
Clinical response				
IFX	-	Ref.	-	Ref.
RTX	-	0.998	-	-
Endoscopic response				
IFX	-	Ref.	-	Ref.
RTX	-	0.993	-	-
Endoscopic remission				
IFX	-	Ref.	-	Ref.
RTX	-	0.999	-	-
Endoscopic improvement				
IFX	-	Ref.	-	Ref.
RTX	-	0.993	-	Ref.

^a^ Adjusted covariates in Multivariate analyses: age-binary variable, sex, BMI-binary variable, disease duration-binary variable, current cigarette smoker, MCS category, Disease extent > proctitis, MCS, MES-binary variable, ALB, FC, CRP, and >1 advanced therapy class failure (biologic or small molecule).

^b^ Adjusted covariates in IPTW cohort: Age, sex, BMI, disease duration, disease extent (>proctitis), MES ≥ 2, PR3-ANCA levels, >1 advanced therapy class failure (biologic or small molecule), and concurrent oral prednisone. All included covariates are binary variables, except PR3-ANCA levels.

### 3.4 Sensitivity analysis

In a sensitivity analysis, baseline MCS, MCS category, ALB, FC, and CRP were added as covariates to the IPTW logistic regression models. The MLRS results were again similar: clinical remission (OR 170, 95%CI: 9.113–1,286.020).

### 3.5 Relationship between PR3-ANCA reduction and clinical remission

Since B lymphocyte levels were not routinely monitored in patients treated with IFX, we were unable to perform dichotomous analysis on B lymphocyte data in this group, nor could we conduct a correlation analysis between B cell reduction rate and PR3-ANCA reduction rate. As shown in Table 4, for every 50% reduction in PR3-ANCA levels, the OR for clinical remission was 7.583 (95%CI: 1.648–34.903). Furthermore, this conclusion remained robust after adjusting for confounding factors.

**TABLE 4:** The relationship between PR3-ANCA reduction rate and clinical remission.

PR3-ANCA reduction rate	OR (95%CI)	*P*
Unadjusted		
≤50%	-	Ref.
>50%	7.583 (1.648–34.903)	0.009
Adjusted^a^		
≤50%		
>50%	25.847 (1.630–409.780)	0.021

^a^ Adjusted covariates in Multivariate analyses: age-binary variable, sex, BMI-binary variable, disease duration-binary variable, current cigarette smoker, MCS category, Disease extent > proctitis, MCS, MES-binary variable, ALB, FC, CRP, and >1 advanced therapy class failure (biologic or small molecule).

### 3.6 Safety

Among the patients, a total of 2 cases showed elevated interferon tests for tuberculosis. None of them were on RTX, while 2 were IFX patients, accounting for 7.69%. There were no patients with elevated EBV-DNA, HBV-DNA, or CMV-DNA levels. Additionally, no severe infections were reported. In addition, the baseline seropositivity rates (HBsAg+) were 0% in the RTX group and 3.85% (1/26) in the IFX group, respectively. Notably, no HBV reactivation or infection occurred in either group. Mean IgG levels remained stable in RTX-treated patients (11.50 ± 3.59 vs. 10.84 ± 1.48, *P* = 0.569).

## 4 Discussion

RTX is recognized as an effective therapy for the treatment of various conditions, including multiple malignant lymphomas (Martin et al., 2007), rheumatoid arthritis (St Clair, 2009), membranous nephropathy (St Clair, 2009), and ANCA-associated vasculitis (Raffray and Guillevin, 2020). Although the B-cell depletion strategy is highly anticipated for treating UC, a clinical trial showed that the CD20-targeting monoclonal antibody rituximab did not improve outcomes in ulcerative colitis, which has tempered enthusiasm for its use in UC patients (Leiper et al., 2011). However, there are significant differences between that study and ours: First, the ANCA status of UC patients was not specified in their research, and their study population likely included both ANCA+ and ANCA-negative individuals. This lack of stratification in their cohort could have led to an underestimation of RTX efficacy. Second, their study administered a single 1,000 mg dose of RTX, which may have conversely exacerbated the disease. Third, no dynamic monitoring of B-cell depletion was conducted in their research, making it unclear whether RTX had depleted B cells to a therapeutically effective level. In this study, we focused on PR3-ANCA + UC patients who received RTX treatment at doses of 100 mg or 200 mg, with administration continued until B-cell depletion was achieved. We present real-world data on the use of RTX for treating moderately to severely active PR3-ANCA + UC patients. To the best of our knowledge, while there are existing studies evaluating rituximab in UC globally, our study is the first in the Hubei region (Wuhan) to report real-world evidence of RTX efficacy and safety in this specific subgroup (PR3-ANCA+, moderately to severely active UC). Additionally, our 22-week follow-up data, obtained from a real-world setting where dosing was individualized based on B-cell depletion status, provide valuable insights into the clinical application of RTX in this local patient population.

The prevalence of PR3-ANCA+ in UC patients varies significantly across different populations: it is 8.6% among Chinese individuals, 39.2% among Japanese individuals, 12.1% among Swedish individuals (Elzouki et al., 1999), and 14.1% among Spanish individuals (García-Herola et al., 1998). In this study, we focused on UC patients who are PR3-ANCA+. Our findings indicate that more than half of the cases presented with severe active UC [66.67% (8/12) in the RTX group and 46.15% (12/26) in the IFX group], and 100% had disease extent beyond proctitis. These results suggest that PR3-ANCA+ is associated with a more severe form of UC (Mahler et al., 2013; Takedatsu et al., 2018; Aoyama et al., 2021; Imakiire et al., 2022; Wen et al., 2022; Zeng et al., 2023).

Two clinical trials have reported on the outcomes of IFX treatment for moderate-to-severe UC patients who are refractory to corticosteroids (Rutgeerts et al., 2005; Jiang et al., 2015). The proportions of clinical remission were 51.20% (21/41) in Jiang XL’s trial and 33.90% (41/121) in the ACT1 trial. Clinical response was seen in 65.8% (27/41) and 52.1% (63/121) of patients, respectively. Endoscopic improvement was noted in 53.7% (22/41) and 50.4% (61/121) of cases at week 30 of IFX treatment. The GRADENIA trial reported that 23% (64/198) of cases achieved endoscopic remission at week 54 (Danese et al., 2022). These proportions are higher than the rates of clinical remission (23.08%, 6/26), endoscopic improvement (34.62%, 9/26), and endoscopic remission (11.54%, 3/26) observed at week 22 in the present study. However, the proportion of clinical response in this study, 65.38% (17/26), was similar to the rates reported in previous studies (Rutgeerts et al., 2005; Jiang et al., 2015). It should be emphasized that all effectiveness endpoints in this study were evaluated at week 22. However, the response to IFX is known to decrease with the duration of drug administration, suggesting that the proportion of clinical response may decrease at week 30 and even more so at week 54. In conclusion, PR3-ANCA + moderately to severely active UC patients tend to have a worse response and effectiveness to IFX (Taylor et al., 2001; Ferrante et al., 2007; Jürgens et al., 2010; Yoshida et al., 2021). The proportions of clinical remission, clinical response, endoscopic response, endoscopic remission, and endoscopic improvement in the RTX group were significantly higher than those in the IFX group before and after IPTW. These results suggest that RTX may be more responsive and effective than IFX in this patient population.

Peripheral blood PR3+ B cells are the primary precursor source of peripheral blood PR3-ANCA, and their levels show a significant positive correlation (Berti et al., 2021). Our results demonstrated that for every 50% reduction in PR3-ANCA levels, the OR for clinical remission was 7.583 (95% CI: 1.648–34.903). Furthermore, this conclusion remained robust after adjusting for confounding factors. This aligns with the pathogenic role of ANCA and supports B-cell targeting as a precision therapeutic strategy.

During the treatment period, there were no cases of elevated interferon tests for tuberculosis in the RTX group (0.00%), and two cases in the IFX group (7.69%). In addition, no HBV reactivation or new HBV infection was observed in either group. For patients receiving RTX treatment, mean IgG levels remained stable (11.50 ± 3.59 vs. 10.84 ± 1.48, P = 0.569). These findings—specifically the absence of HBV reactivation and hypogammaglobulinemia—support the safety of RTX in Asian UC populations, where HBV is endemic. The real-world application data of RTX indicates a reliable safety profile; however, the presence of latent infections and the risk of their activation still require support from long-term follow-up data.

It is important to note the limitations of this study: (1) The retrospective nature of the study may introduce selection bias in subject selection. (2) The sample size is small, which may affect the generalizability of the results. (3) The follow-up time is short, and the long-term effectiveness of the treatment remains unclear. These constraints underscore the necessity for additional studies with expanded cohorts and extended observation times to more thoroughly assess the effectiveness and safety of Rituximab (RTX) in treating patients with moderate-to-severe ulcerative colitis who are PR3-ANCA+.

In conclusion, RTX was more effective than IFX in this patient population, with a similar safety profile. In clinical practice, RTX may be considered by physicians when IFX are ineffective. However, further follow-up is needed to observe the long-term effects of RTX. Additionally, prospective, double-blind, randomized controlled trials are necessary to provide definitive indications for the use of RTX in UC patients with PR3-ANCA+.

## Data Availability

The raw data supporting the conclusions of this article will be made available by the authors, without undue reservation.
