# Supplementary material for: Rituximab in PR3-ANCA positive patients with moderately to severely active ulcerative colitis: a multicenter real-world pilot study
Source: Front Pharmacol. 2025 Oct 24;16:1621795. doi: 10.3389/fphar.2025.1621795 (PMC12592138; doi:10.3389/fphar.2025.1621795)
Supplement: Supplementary file 1 [file Supplementaryfile1.docx]

**Supplementary Table 1 The definition of endpoints**

| Endpoints | Definition |
| --- | --- |
| Clinical remission | MCS of ≤2 with individual subscores of ≤1 |
| Clinical response | At least a 3-point decrease and at least a 30% reduction in MCS from baseline, plus at least a 1-point decrease in rectal bleeding subscore or an absolute rectal bleeding score of 0 or 1 |
| Endoscopic response | At least a 2-point decrease in MES from baseline |
| Endoscopic improvement | MES of ≤1 |
| Endoscopic remission | MES of 0 |

Abbreviations: MCS, Mayo Clinic Score; MES, Mayo Endoscopic Subscore

**Supplementary Table 2 Percentage of B lymphocytes and PR3-ANCA levels in patients receiving RTX therapy**

| Number | B lymphocytes(%) | | | | | PR3-ANCA(CU) | | | | |
| --- | --- | --- | --- | --- | --- | --- | --- | --- | --- | --- |
|  | Baseline | Week 2 | Week 6 | Week 14 | Week 22 | Baseline | Week 2 | Week 6 | Week 14 | Week 22 |
| Patient 1 | 11.97 | 0.02 | 0.00 | 0.06 | 0.06 | 277.60 | 203.50 | 83.70 | 34.80 | 25.90 |
| Patient 2 | 7.25 | 0.08 | 0.08 | 0.42 | 0.20 | 36.50 | 46.50 | 20.50 | 7.30 | 4.00 |
| Patient 3 | 22.81 | 0.06 | 0.11 | 0.14 | 0.30 | 63.50 | 50.30 | 31.10 | 16.30 | 15.30 |
| Patient 4 | 15.81 | 0.02 | 0.02 | 0.02 | 1.75 | 44.60 | 43.50 | 38.90 | 36.50 | 32.40 |
| Patient 5 | 1.19 | 0.10 | 0.80 | 0.00 | 0.04 | 324.90 | 256.50 | 188.30 | 153.00 | 127.00 |
| Patient 6 | 12.27 | 0.02 | 0.02 | 0.02 | 0.04 | 134.80 | 90.70 | 46.80 | 22.70 | 15.60 |
| Patient 7 | 4.91 | 0.02 | 0.00 | 0.06 | 0.02 | 36.90 | 11.70 | 7.10 | 5.90 | 3.00 |
| Patient 8 | 4.47 | 0.86 | 0.25 | 0.10 | 0.04 | 77.70 | 64.00 | 50.30 | 50.30 | 27.80 |
| Patient 9 | 9.80 | 0.00 | 0.06 | 0.10 | 0.30 | 54.90 | 51.20 | 39.80 | 32.50 | 24.60 |
| Patient 10 | 23.64 | 0.02 | 0.00 | 0.96 | 0.55 | 104.70 | 109.05 | 82.00 | 55.20 | 33.30 |
| Patient 11 | 25.89 | 0.02 | 0.22 | 0.80 | 7.30 | 210.10 | 99.00 | 63.30 | 27.60 | 21.90 |
| Patient 12 | 9.36 | 0.29 | 0.02 | 0.22 | 0.18 | 361.10 | 211.60 | 142.40 | 138.90 | 109.70 |

**Supplementary Table 3 The efficacy endpoints in patients receiving RTX Therapy**

| Number | Efficacy endpoints at week 22 | | | | |
| --- | --- | --- | --- | --- | --- |
|  | Clinical remission | Clinical response | Endoscopic response | Endoscopic remission | Endoscopic improvement |
| Patient 1 | ✔ | ✔ | ✔ | ✔ | ✔ |
| Patient 2 | × | ✔ | ✔ | × | ✔ |
| Patient 3 | × | ✔ | ✔ | × | ✔ |
| Patient 4 | ✔ | ✔ | ✔ | ✔ | ✔ |
| Patient 5 | ✔ | ✔ | ✔ | ✔ | ✔ |
| Patient 6 | ✔ | ✔ | ✔ | ✔ | ✔ |
| Patient 7 | ✔ | ✔ | ✔ | ✔ | ✔ |
| Patient 8 | ✔ | ✔ | ✔ | × | ✔ |
| Patient 9 | ✔ | ✔ | ✔ | ✔ | ✔ |
| Patient 10 | ✔ | ✔ | ✔ | × | ✔ |
| Patient 11 | ✔ | ✔ | ✔ | × | ✔ |
| Patient 12 | × | ✔ | ✔ | × | ✔ |
